# A framework for planning and facilitating video-based professional development

**DOI:** 10.1186/s40594-017-0086-z

**Published:** 2017-11-21

**Authors:** Miray Tekkumru-Kisa, Mary Kay Stein

**Affiliations:** 1 School of Teacher Education & Learning Systems Institute, Florida State University, Tallahassee, FL USA; 2 Learning Research and Development Center, University of Pittsburgh, Pittsburgh, PA USA

## Abstract

**Background:**

Recent transformative changes in science education require new learning opportunities for teachers—opportunities that include rich images of classroom enactment of the reform vision. One fruitful way for doing that is to use video clips of instruction.

Teachers do not, however, learn how to improve their instructional practice from simply watching and reflecting on classroom videos. The videos need to be carefully selected and embedded in professional development in ways that—through facilitator-led, participant-centered discussion—can help teachers to notice and reason about important aspects of instruction and learning that occur in the video. Consistent with the recent efforts to identify planning and facilitation approaches that guide effective professional development (PD) programs, in this paper, we adapted the Five Practices Framework for orchestrating productive classroom discussions to describe how PD facilitators plan for and enact professional learning tasks to help science teachers learn within a video-based PD program. These practices include anticipating, sequencing, monitoring, selecting, connecting and two additional practices that set the stage for the five practices (i.e., setting goals and selecting tasks).

**Results:**

Our analyses of the video-based discussions in the PD provide insights into how the facilitators engaged teachers in video-based conversations by using the practices of monitoring, selecting, and connecting. The monitoring moves, such as clarifying, countering, and redirecting, were used by the facilitator in nearly all the PD sessions. Similarly, selecting moves were used and were consistent with the goals of the PD. Finally, analysis of facilitators’ and participants’ connecting comments indicated their increased capacity to make connections to the bigger ideas of teaching science by maintaining the cognitive demand on students’ thinking.

**Conclusions:**

This paper provides elaborated descriptions of the five practices for planning and facilitating video-based PD and the ways in which they were enacted in a video-based PD program in science. In so doing, it proposes five practices as a guiding framework to support teachers’ learning from videos. Overall, the study’s results endorse the promise of a goal-driven, theory-informed design that foregrounds careful attention to teachers’ thinking in ways that support their understanding of complex classroom interactions.

**Electronic supplementary material:**

The online version of this article (doi:10.1186/s40594-017-0086-z) contains supplementary material, which is available to authorized users.

## Background

The Framework for K-12 Science Education (NRC, 2012) and the Next Generation Science Standards (NGSS Lead States, 2013) bring a new vision for science teaching and learning. This vision is based on key findings from research (NASEM, 2015) such as integrating knowing and doing is key for science learning (e.g., Pickering, 1992) and developing understanding through engagement in scientific practices is more productive for future learning than memorizing facts (e.g., Cognition and Technology Group at Vanderbilt, 1993). The new vision represents a significant departure from how most students currently learn science (Banilower et al. 2013; Reiser, 2013). For example, based on an observational study of a nationally representative sample of schools, a common weakness in observed science lessons was the opportunities for student sensemaking (NASEM, 2015; Weiss et al. 2003).

As with other transformative reforms, teachers will need access to carefully designed professional development (PD) (Wilson, 2013) to help them learn how to support students’ sensemaking in science classrooms. Such PD would benefit from opportunities for teachers to analyze and discuss images of teaching as they try to enact the reform vision in their classrooms (Borko et al., 2011; NRC, 2015; Reiser, 2013). An increasingly popular way of doing this is through the use of video clips of classroom interactions that can create opportunities for analysis that digs beneath the surface of reforms (Reiser, 2013; Sherin & Han, 2004; van Es & Sherin, 2007).

Research conducted over the past decade suggests that video can be a powerful tool for facilitating teacher learning (Borko et al., 2008; Borko et al. 2011; Gaudin & Chalies, 2015; Moon & Michaels, 2016; Roth et al. 2011; Sherin, 2004). As a particular manifestation of practice-based PD (Ball & Cohen, 1999; Gallucci et al., 2010; Loucks-Horsley et al. 2010), *videos* focus teachers’ learning on real classrooms and capture teaching in all of its complexity while, at the same time, affording space and time for reflection (Fishman et al., 2014; Miller & Zhou, 2007; Sherin, 2004; Zembal-Saul, 2008).

Teachers do not, however, learn how to improve their instructional practice from simply watching and reflecting on classroom videos (Brophy, 2004). As discussed by others, videos are simply tools for supporting teacher learning in PD settings like manipulatives or laboratory materials are tools for facilitating students’ mathematical and scientific understanding in the classroom (Goldsmith & Seago, 2011; van Es et al., 2015). To get the most out of video-based PD, the structures and tasks designed around the video clip should be considered carefully (Brophy, 2004; Le Fevre, 2003; Seidel et al. 2005). The videos need to be carefully selected and used in ways that—through facilitator-led, participant-centered discussion—help teachers to notice and reason about important aspects of instruction and learning that occur in the video (Borko et al. 2008; Sherin et al., 2009; Zhang et al., 2011).

Recent studies, mostly in mathematics education, have begun to recognize and emphasize the role of the facilitator to assure that teachers benefit from the specific affordances of videos (e.g., Borko et al., 2014a; van Es et al., 2014; Zhang et al. 2011). In fact, as stated by Borko et al. (2014b), “presently in the United States, PD facilitators represent a new cadre of prominent players on the educational scene” (p. 149). As effective PD programs “scale up” in order to reach more and more teachers, teacher leaders, coaches, and administrators are playing critical roles as facilitators of video-based PD (Maarongele et al. 2013). Given their key role, there is an urgent need to help these facilitators learn how to plan and facilitate newly developed and effective PD programs that use video as the main artifact of practice (Borko et al. 2014a). Indeed, a report by the National Academies of Sciences, Engineering, and Medicine on strengthening science education through a teacher learning continuum (2015) concluded that the development of science teacher leaders can be an important mechanism to support ambitious science instruction. One of the report’s key recommendations was the need to support ongoing teacher learning through the design and implementation of “research focused on the learning needs of teacher leaders and professional development providers” (p. 231).

Consistent with recent efforts to identify approaches for planning and facilitating effective PD programs, the purpose of this paper is to propose a framework for planning for and facilitating video-based PD and also to examine the design and facilitation of a particular video-based PD program in science through the lens of this framework. In doing so, we argue that frameworks can play a critical role in helping PD leaders/facilitators learn how to plan for and implement effective PD sessions designed around videos of teaching and learning in science classrooms. As noted by Smith (2014), tools can provide a scaffold for teacher learning, “a structure that allows them [teachers] to do something that would otherwise be challenging or impossible to do” (p. 3). Moving one layer up, we argue that PD facilitators can also benefit from tools and frameworks that enable them (the PD facilitators) to do something that otherwise would be challenging to do. In short, using frameworks can help to guide PD facilitators’ thinking and practices as they plan for and orchestrate discussions around video clips of instruction within PD sessions.

In this paper, we focus on how to plan for and facilitate productive discussions around carefully selected video clips of science instruction to support teachers’ learning. After briefly introducing the Five Practices Framework, the paper unfolds in four sections. In the first section, we describe the context and structure of the particular video-based PD program (Teaching Science with Cognitive Demand (TSCD) on which we focus as we describe and use the Five Practices Framework. Although the Five Practices Framework provides us a lens to examine the design and enactment of any video-based PD program, here, we illustrate its use—in very concrete and tangible ways—with a particular PD program, TSCD. In the following sections, we unpack each practice, first by conceptually describing it, then by elaborating the role that it played in the design and enactment of the TSCD-PD. The second section introduces practices that are related to *planning* for video-based PD sessions. In the third section, we introduce the remainder of the practices, which are related to *facilitating* PD sessions. To illustrate how these facilitation practices were enacted in the TSCD-PD, we also report the results of an empirical analysis of the moves made by the facilitators as discussions actually unfolded over the six TSCD-PD sessions during which videos were used. In the final section, we discuss the utility of frameworks for guiding inquiry into preparing for and facilitating video-based PD, addressing the role played by frameworks in both highlighting and potentially obscuring important elements of a successful PD program.

### *Five Practices Framework* for planning and facilitating video-based PD

The framework, first used at the classroom level in mathematics (Stein, Engle, Smith, & Hughes, 2008; Smith & Stein, 2011), identifies approaches to the preparation and facilitation of video-based PD during which student reasoning and teachers’ instructional practices are examined. We are not the first to appropriate classroom-based frameworks and tools to professional development settings. In particular, PD leaders and researchers are increasingly turning to the literature on classroom-based teaching and learning for ideas on how to manage discussions (i.e., Borko et al. 2014b; Elliott et al. 2009; van Es et al., 2014).

PD facilitators can be teacher leaders, coaches, district leaders, researchers, or other educators. However, in the PD context, they all become *teachers of teachers*. Even though learning to teach teachers is different from learning to teach, they are also related (NASEM, 2015). For example, facilitators and teachers who ascribe to a learner-centered approach face similar challenges. Teachers need to learn how to orchestrate whole-class discussions that use a wide array of student responses to instructional tasks and productively build on them in ways that advance students’ disciplinary understanding (e.g., Ball, 1993; Lampert, 2001). Similarly, in video-based PD, facilitators are faced with a wide variety of teacher responses to the video (as it is an open-ended task) and the facilitator must carefully listen to, interpret, and gently shape those responses toward a productive end. The critical idea (in both classroom- and PD-based discussions) is to support learning toward carefully defined goals without undermining learners’ sense of agency. To be sure, there are differences as well. For example, facilitators may face special challenges when pressing teachers’ thinking in PD settings because it requires being critical. This may “run counter to most teacher PD in which politeness is valued” (Jackson et al. 2015, p. 95) and in which the facilitator is sometimes a peer, or even less-experienced than the teachers s/he is teaching.

Others have also proposed frameworks for facilitating video-based PD discussions inspired by the Five Practices (e.g., Borko et al. 2014b; Elliot et al. 2009). For example, one of the components of Lesseig et al. (2016) design framework for leader professional development was practices for orchestrating mathematical discussions. They stated, “similar to the teacher’s role in classrooms, we contend that leaders should also be strategic and thoughtful in how they anticipate, monitor, select, and sequence teachers’ sharing of their solutions.” Here, we build on and extend their work by further adapting the Five Practices Framework to video-based PD research and at the same time by extending it to science education. More specifically, we have adapted the Five Practices to describe how *PD facilitators* plan for and enact professional learning tasks to help *science* teachers learn within a video-based PD setting.

### *Teaching Science with Cognitive Demand* video-based professional development

TSCD video-based PD was designed to improve K-12 practicing science teachers’ capacities to use cognitively demanding tasks effectively by maintaining high levels of student thinking and reasoning in their classrooms. TSCD-PD was situated within the context of an NSF-funded project that developed biology units aligned with the NGSS (Schunn & Stein, 2009). The participants^1^ were implementing one of these biology units, which was about Mendelian Inheritance (hereafter referred to as the Design Unit) during the same period of time as they were participating in the PD. Thus, they all had access to cognitively demanding instructional tasks and these form the basis of video clips that were discussed in the TSCD-PD (see Additional file 1 for an example). The video-clips used in the PD were selected from prior implementations of the Design Unit in high-school biology classrooms.

Five biology teachers (Table 1) from several different school districts in the northeastern region of the US voluntarily participated in the PD and were paid for their participation.

**Table 1:** Information about the participants of the study

Teacher (*pseudonym*)	Information about the teacher			Information about the classroom	
	Years teaching	Years teaching biology	Bachelor’s degree	Grade level	School percentage of free and reduced lunch
Carol	5	2	Biology	12	*Not available*
Linda	16	16	Biology	10	35%
Susan	13	13	Biology	10	35%
Barbara	3	2	Biology	11	63%
Nancy	3	2	Biology	11	82%

As summarized in Table 1, all the PD participants were teaching biology at different high-school grade levels. Their experience levels varied, as did the SES status of their students. Linda and Susan were the two most experienced teachers from the same public school where about 35% of the students were eligible for free and/or reduced lunch. Other teachers were in their early years of teaching biology. Linda was the only teacher in the PD who had prior experience in implementing the Design Unit. Carol taught at Catholic school. Like Linda, Carol had experience in working with the project team, but on a different biology unit. Barbara and Nancy were from two different schools operating under the same charter school organization, which focused on the use of research-based practices in the classroom. Both of these schools have a high proportion of students eligible for free and/or reduced lunch.

The TSCD-PD took place once or twice a week over a 1-month period for a total of seven meetings, and each of which was 3 h in duration. Each TSCD-PD session was organized into two main parts: (1) analysis of science instructional tasks as presented in written materials and (2) discussion of a video case that illustrated the classroom enactment of a high-level task, most typically one of the tasks from the Design Unit that was just analyzed.^2^

Previous studies of TSCD-PD have found positive impacts on teachers’ learning to notice as well as impacts on their instructional practices. More specifically, analysis of interviews before and after TSCD-PD, during which teachers were asked to respond to novel video clips presenting the enactment of cognitively demanding biology tasks, indicated an increase in teachers’ tendency to notice important features of classroom interactions. Teachers began to see teaching as constituted in the interaction of the teacher, students, and task (as opposed to a view of teaching as a solo act) (Tekkumru-Kisa & Stein, 2015a). In addition, analyses of instructional data collected from teachers’ classrooms before and after the PD have provided evidence for changes in teachers’ instructional practices associated with facilitating high-level student thinking such as attending to and advancing students’ thinking and pressing students for sensemaking (Tekkumru-Kisa & Stein, 2015b).

### Unpacking the Five Practices Framework: Planning for productive video-based PD

Just as teachers’ preparation for lessons that involve discussion will benefit from careful planning, we conjecture that facilitator preparation for PD will lead to richer, more coherent discussions. And, similar to Five Practices for mathematics teaching, facilitators may want to consider two additional practices that “set the stage” for the other five: Setting goals and selecting tasks.

### Laying the groundwork: Setting goals and selecting tasks

#### Setting goals

For teachers, setting a goal that reflects an important, to-be-learned disciplinary idea or insight is a key. “Goals set the stage for everything else” (Hiebert et al. 2007, p. 51).

We have found that determining the goal for teacher learning is a helpful practice for PD facilitators to embrace as well. Being clear about what participants will learn can help with not only the design but also the enactment of the PD. Goals can serve as reference points for “sizing up” and guiding discussions about the videos. When used by skilled facilitators, they can become the “north star” by which to steer participants’ contributions toward a deeper understanding of, in the case of TSCD-PD, how to maintain students’ thinking and reasoning at high levels. The importance of goal-setting is increasingly recognized (e.g., Borko et al. 2014b; Jackson et al. 2015; van Es et al. 2015). Prior research on teachers’ learning to notice from the videos, for instance, emphasizes establishing clear goals and specific prompts to guide discussion (e.g., Goldsmith & Seago, 2011; Santagata, 2011). Similarly, Lesseig et al. (2016) included identifiying a mathematical goal for teacher learning in the framework that they proposed for leader professional development. In many of these PD studies, teachers work on mathematical tasks. Therefore, there seems to be a growing agreement in the literature on the importance for setting clear goals for teachers’ learning, but the nature of goals differs depending on the professional development and its overall purpose. Overall though, research is silent on examining how PD facilitators *use* goals as a framework for interpreting participants’ contributions and deciding which to highlight for further discussion or elaboration as a way to move the entire groups’ developing understandings in productive directions.

The design of the TSCD-PD was driven by a desire to make the video-based discussion productive by focusing on clear goals for teachers’ learning. For us, discussions are productive when they both build on participants’ ideas *and* lead to an intended, valued outcome that is aligned with a pre-determined goal. Our overall goal for the set of TSCD-PD sessions was to develop participants’ capacity to select and successfully enact cognitively demanding tasks in their classrooms by maintaining high-level demand on students’ thinking and sensemaking. Toward that end, we aimed to support participants’ learning to notice classroom interactions in different ways as well as identify and use a set of instructional practices (referred to as the “factors”; Stein, Grover, & Henningsen, 1996) that research suggests help to maintain high levels of student thinking and reasoning while students work on cognitively demanding tasks. The means of accomplishing these goals consisted primarily of participants’ watching selected video clips of teaching and discussing what they viewed.

In the design of TSCD-PD, we also identified subgoals involved in reaching the overall goal. For example, prior research has found that moving away from a nearly exclusive focus on the teacher in the video to beginning to notice and make sense of students’ thinking is an important positive shift in how teachers learn from video (e.g., Sherin & Han, 2004; van Es & Sherin, 2007). In addition, logically, participants would need to learn how to identify student thinking associated with various levels of cognitive demand.

#### Identifying professional development tasks

For teachers, the next step after identifying what they want their students to learn is to design or select an instructional task that has the potential to surface the disciplinary ideas or relationships that they want their students to learn. Different tasks provide different opportunities for students’ learning, and it is important that teachers are cognizant of them while selecting tasks for the lesson.

For video-based PD, the choice of a professional learning task is also a key. Prior research has cautioned that in order to get the most out of what videos afford, the structures and tasks designed around video clips should be considered carefully (Brophy, 2004; Le Fevre, 2003). Like classroom tasks, different PD tasks provide different opportunities for teachers to learn about classroom interactions and instructional practices.

The overall design of the learning experiences in the TSCD-PD was based on theories of learning. Each video discussion was conducted in a learner-centered manner while, at the same time, guiding teachers to construct new understanding of teaching (Bransford et al., 1999; Loucks-Horsley et al. 2010). As suggested by Loucks-Horsley et al. (2010), TSCD-PD facilitators guided PD participants “to construct knowledge in the same ways as do effective learning experiences for students” (p. 76). Because the videos represented the authentic work of teaching, the PD tasks in TSCD-PD were ideally suited to this. In such learning environments, the task must be rich enough to support sustained reasoning and productive struggle.

Also embedded within TSCD-PD is the notion that participants’ movement from initial, unformed ideas toward understanding consistent with the goals of the PD must be carefully scaffolded (In TSCD-PD, this scaffolding happened through the “selecting” and “connecting” practices, which will be discussed below). The design of TSCD-PD is also grounded in a view of learning that takes into account the contexts within which people interact as well as their access to resources such as ambitious curriculum materials (Greeno, 2006). This perspective was especially useful for the design of teacher learning situations that provided opportunities for teachers working together to collectively reflect on artifacts of practice (Ball & Cohen, 1999, Borko and Koellner, 2008, Fishman & Davis, 2006; Putnam & Borko, 2000). Therefore, within professional development settings, teachers’ learning is considered to be the result of participation in joint activities to which teachers bring varying levels of expertise (Borko et al. 2008; Greeno, 1997; Lave and Wenger, 1991) and focus on artifacts of practice, which make the teaching a central focus of professional learning (Ball & Cohen, 1999; Borko & Koelner, 2008; Putnam & Borko, 2000).

In each session, the PD tasks around the videos were carefully designed. The selection of the videos was guided by research, which indicates that the cognitive demand of tasks can change as they pass from written materials to how they are set up by the teacher in the classroom to how they are actually enacted or carried out by students and the teacher (Stein et al., 1996). For the TSCD-PD, we identified four video cases that represented either the maintenance or decline of high levels of cognitive demand from written materials to how the task was enacted in the classroom. The Task Analysis Guide in Science (TAGS) (Tekkumru-Kisa, Stein, & Schunn, 2015) was used to differentiate the level and type of student thinking that was occurring in the video case. The TAGS is a two-dimensional framework that can be used to analyze the level and kind of student thinking. The integration/isolation dimension identifies whether or not science content and scientific practices are integrated within a task. The second dimension of the TAGS is *cognitive demand*, which is defined as the level of thinking required of students to complete a particular activity (Doyle, 1988).

Every video was selected to represent a case of a particular level and kind of student thinking during the implementation of a cognitively demanding science task. In session 2, participants first analyzed a video showing a task that was set up (introduced to the students) at a high level of cognitive demand. This was followed by a video in which that same task—same classroom, same lesson—was enacted. In this case, when students actually went about working on the task, their thinking was very shallow and focused on the scientific terminology at a superficial level. In the third TSCD-PD session, participants, once again, analyzed a video showing a high-level set up, but of a different science task. Then, they viewed and discussed two different video clips, both of which represented high-level student thinking as students worked on this cognitively demanding task in the same classroom.

The design of the PD tasks in the fourth and fifth TSCD-PD sessions was again guided by research; this time, it was undergirded by a line of research on learning from contrasting cases (e.g., Garner, 1974; Gibson, 1969; Bransford & Schwartz, 1999). Participants were shown, in succession, two videos that featured the same high-level task. In one video, the teacher moves were very directive and the level of cognitive demand in student thinking declined. In the other video, the teacher maintained the level of cognitive demand through the use of several of the instructional factors associated with maintaining cognitive demand on student thinking. This task is an example of a PD task that combined learner struggle followed by a more direct facilitation style. This cognitive psychological research suggests that contrasting cases help to make particular aspects and dimensions of cases more salient and differentiated from others. As demonstrated by Schwartz and Bransford (1998), who viewed contrasting cases as a way to provide preparation for future learning, initial struggle with a complex task can set up a learner’s receptivity to learning from direct telling. In our case, after struggling to discern the similarities and differences between the two videos, the participants listened to a “mini-lecture” in which the facilitator explicated the similarities and differences between the video cases by using the language associated with factors of maintenance and decline.

### Practice 1: Anticipating responses

One of the reasons that the use of complex tasks in teaching and learning environments is challenging is because high-level tasks are likely to elicit divergent ways of entering and making sense of the task. When teachers prepare for lessons by anticipating how students will approach such tasks, they are not caught flat-footed by novel approaches or ideas. Anticipating students’ responses involves more than assessing whether the students will find the task easy or challenging and interesting or not. It involves developing expectations regarding possible ways in which students might interpret and solve a problem and the array of possible strategies students may use to tackle the problem.

Similarly, facilitators can prepare for a PD session by anticipating how participants might think about and respond to the selected video clip; in doing so, they will be better prepared to deal with “off-track” responses and to make the most of partially formed responses that reveal positive shifts in how participants are thinking.

In the case of TSCD-PD, the facilitator reviewed the literature on teachers’ learning to notice (e.g., Sherin & Han, 2004; van Es and Sherin, 2007) and identified findings related to how teachers typically analyze video clips in early PD sessions (e.g., focus on the teacher in the video, make evaluations). This knowledge, combined with the explicit goals for the TSCD-PD, supported the facilitator as she prepared for each session by watching the video and reading the transcript of the lesson. This knowledge also allowed the facilitator to prepare ways to prompt teachers’ thinking ahead of time instead of only in the flux of a fast-paced discussion. The facilitator also prepared a set of questions that she could ask the participants during the discussion.

### Practice 2: *Sequencing* of videos and PD tasks structured around the videos

The sequencing of the selected pieces of student work during the whole-class discussion is geared toward making the sharing of student work as helpful to as many students as possible. For example, the teacher might begin with student work that represents the most common way that students approached the task. Or, she might begin the discussion with a piece of work that encapsulates a common misconception so as to quickly put the misconception to rest and free up students for listening to and absorbing more promising approaches. The final piece of shared work might represent the most innovative—but most difficult to understand—approach. In short, sequencing can be viewed as way to build from the most easily accessible to the most challenging—but most robust—way to think about the problem and the ideas embedded in it.

The sequencing of videos and professional learning tasks was also based on how we expected the participants to respond to them and a rough trajectory of the road we expected them to travel toward reaching the overarching goal of the PD programs. In comparison to sequencing students’ ideas during the instruction in the classroom, sequencing decisions happen in advance in the design of professional development: *Before* the actual PD session. More specifically, the sequencing practice refers to PD facilitators’ sequencing of videos and the related PD tasks within and across the PD sessions by considering the trajectory of ideas that will be advanced throughout the PD.

As noted earlier in our discussion of theory, the role of the TSCD-PD facilitators is not to explicitly teach the instructional factors associated with maintenance of cognitive demand. Facilitators do not provide a list of factors associated with maintenance and decline of student thinking and then ask participants to memorize or apply them. Rather, the PD experience was designed to have these factors surface from participants’ own analysis and discussion of the video clips. The sequencing of PD tasks aimed to allow learner-centered exploration, especially in the beginning sessions. At the same time, we recognized the need for building a common language, for summing up ideas at critical points in time, and for the subtle guidance of participants’ responses toward the intended goal of the discussion. Therefore, through supporting teacher autonomy and exploration with careful and thoughtful scaffolding, we aimed to design a system that provided for teacher agency yet still allowed the facilitators to steer participants toward understanding important issues of teaching and learning consistent with the goals of the TSCD-PD.

We accomplished this largely through detailed consideration of how we would sequence the video-based PD tasks. Specifically, they were sequenced to foster *sensing*, *surfacing*, and *labeling* of instructional factors that help teachers to maintain high levels of student thinking while students are working on cognitively demanding tasks. In the beginning of the TSCD-PD, we felt it was important to allow participants time to become accustomed to the idea of levels of cognitive demand and the different ways that classroom-based enactment of high-demand tasks might look without focusing on terminology. We refer to TSCD-PD sessions 2 and 3 as allowing teachers to *sense* that teachers’ instructional practices, in addition to what the instructional task demands, can catalyze different levels of student thinking and that this might be a novel and productive way to think about instruction.

**Fig. 1:**
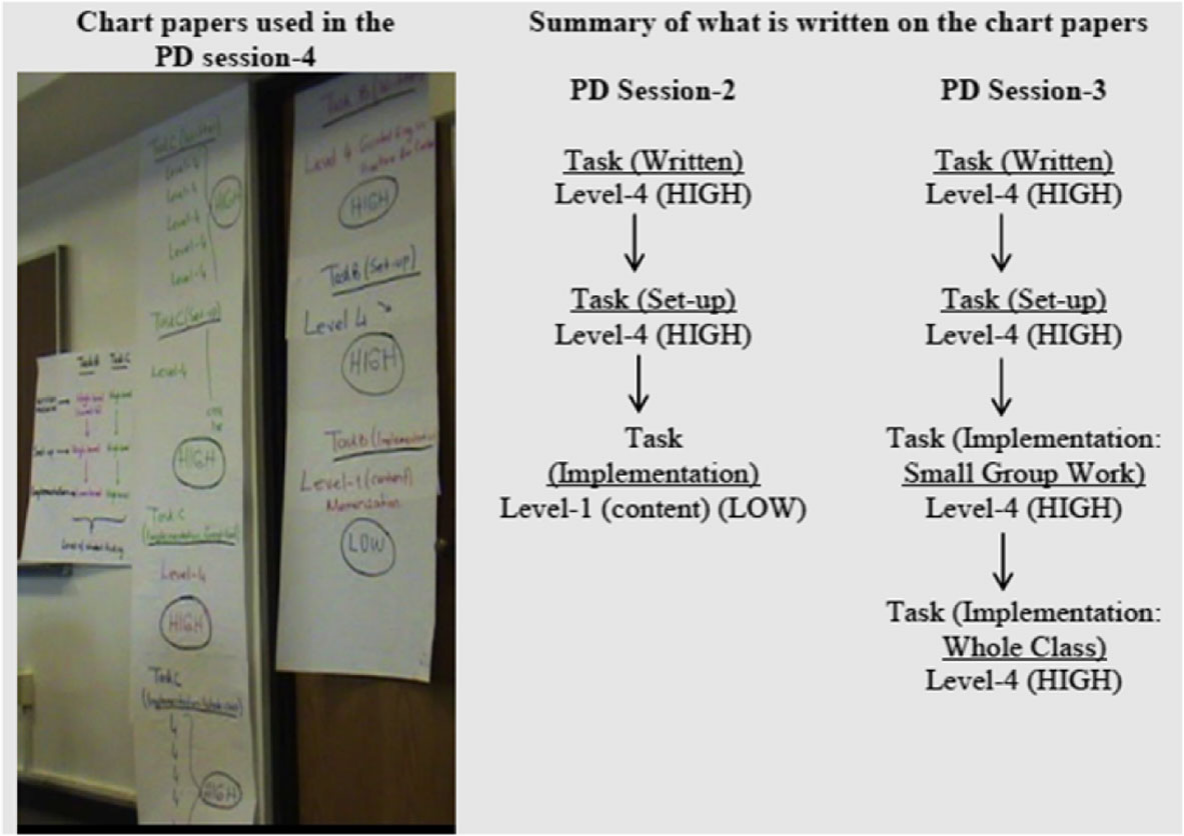
Chart papers from the fourth TSCD-PD session

We started the fourth TSCD-PD session by introducing participants to the key idea behind the mathematical task framework that tasks can change in their level of cognitive demand as they pass from written materials to how they are set up by the teacher in the classroom to how they are actually enacted or carried out by the students. Before viewing the contrasting video cases, the facilitator highlighted this key idea by referring to chart papers that had been produced during sessions 2 and 3 (see Fig. 1). Recorded on the chart paper were PD participants’ agreement of the cognitive demand levels of the main task that was analyzed (1) as it appeared in the written materials, (2) as set up by the teacher in the video case, and (3) as enacted by the teacher and the students during instruction. After making this summary, the facilitator made a short presentation that described the “journey of a task” as presented in the mathematical task framework (Stein et al., 1996). The presentation of the “journey of a task” helped to frame PD participants’ viewing of and the discussions about the contrasting video cases.

This introduction of the contrasting cases in session 4 marked a more concerted effort to *surface* the similarities and differences in classrooms in which cognitive demand was kept at a high level versus declined. We designed the contrasting video cases to support PD participants to notice things like the level and type of student thinking and how the teacher’s pedagogy differentially shaped it (see Tekkumru-Kisa & Stein, 2014, for a more detailed analysis of the contrasting case design and its impact on teacher noticing). The design of this PD task aimed to allow participants to uncover how different teacher moves in the two videos could shape the decline or maintenance of level of student thinking differently.

The remaining sessions involved more explicit naming of the factors that were identified in the contrasting videos and participants’ initial halting attempts to use those *labels* to describe their own interactions with students in their own teaching videos. Specifically, in the beginning of the sixth PD session, participants were engaged in a brief “summary task,” which allowed them to debrief on what they had done/learned about until that session with the task analysis and video analysis. The facilitator, again, referred to all of the chart papers generated in the earlier PD sessions to remind participants what they had done/discussed in the earlier sessions. During this discussion, the facilitator introduced the term “factors associated with maintenance and decline” to the participants once they started to talk about the “reasons” of maintenance and decline.

To derive the factors, participants initially drew on their own experiences or video observations and identified factors that were beyond their control, such as student absenteeism and class size. At this point, the facilitator began charting all of these factors that participants were generating. The discussion then turned to factors that they could exert some control over (things they had observed in the videos or experienced teaching the Design Unit tasks in their own classrooms), such as the nature of teacher questioning. All of these factors were listed on the chart paper; in other words, they were *labeled* for participants to draw on while they were analyzing video clips from their own classrooms. The labels were also populated with additional factors as they analyzed their own videos in these last two TSCD-PD sessions.

### Facilitating productive video-based PD

While the first two practices (i.e., anticipating and sequencing) of the Five Practices Framework occur during the planning phase for video-based PD, the remaining three practices (i.e., monitoring, selecting, and connecting) are related to facilitating productive video-based discussions *during* the PD sessions. In what follows, we will provide a detailed description of these three facilitation practices. Moreover, we will also provide an analysis of the actual video-based discussions that unfolded over the TSCD-PD sessions in which videos were used. It will provide insights into how the facilitators engaged teachers in the video-based conversations by using the practices of monitoring, selecting, and connecting.

### Data sources and analysis of the TSCD-PD sessions

For our empirical analysis, we used transcripts of video records of six TSCD-PD sessions. Each session was about 3 h, approximately 2 h of which consisted of discussion around the videos. The first author was the facilitator of all the PD sessions. The second author mostly played a participant observer role and occasionally supported the facilitation of the sessions by interjecting comments or questions into the discussions. Thus, her comments were also coded as “facilitator moves” in our analysis. A mathematics teacher and two other researchers, who were part of the larger project, also attended some of the sessions and from time to time made comments. These two researchers’ comments were not coded as facilitator moves.

To code the participant discussions, we used van Es et al.’s (2014) framework for facilitating video-based professional development. van Es and colleagues identify more micro facilitator moves associated with engaging teachers in substantive discussion of video in a group setting. We elaborated on the van Es and colleagues’ framework by grouping relevant moves under the larger umbrella of *monitoring* and *selecting* practices. More specifically, we grouped van Es’s codes of clarifying, countering, redirecting, linking participant ideas, distributing participation, and validating participant ideas under *monitoring* and highlighting, lifting up, and pressing under *selecting* (see Table 2). Combining the van Es et al. and the Five Practices Frameworks allowed us to interpret the nature of facilitator’s moves and ideas embedded in the discussions in relation to the goals of the TSCD-PD.

**Table 2:** Grouping of facilitator moves under “monitoring” and “selecting” practices

	Facilitator moves in van Es et al. (2014) framework	Definitions^a^
Monitoring	Countering	Offer an alternative point of view
	Clarifying	Restate and revoice to ensure common understanding of an idea
	Redirecting	Shift the discussion to maintain focus on the task of video analysis
	Linking participant ideas^b^	Make connections between ideas raised in the discussion
	Validating participant ideas	Confirm and support participant contributions
	Distributing participation	Invite participants to share different ideas based on who is (and is not) participating [and asking for more ideas such as “Anything else?”]
Selecting	Pressing	Prompt participants to explain their reasoning and/or elaborate on their ideas [and also asking for evidence]
	Lifting up	Identify an important idea that a participant raised in the discussion for further discussion
	Highlighting	Direct attention to noteworthy student ideas in the videos

^a^These definitions are directly taken from van Es et al. (2014). In our analysis, we expanded the definitions of some of the moves as indicated in brackets

^b^We changed the name of the move “connecting ideas” in van Es et al.’s framework to “linking participant ideas” to eliminate confusion between this move and “connecting” practice of the Five Practices Framework

*Selecting moves* were coded in two phases. Specifically, when a facilitator move was coded as:

- *Pressing*, we also identified the ideas that the facilitator prompted participants to expand on or to further their reasoning about
- *Lifting up*, we also identified the ideas that the participants raised in the discussion that the facilitator elevated
- *Highlighting*, we also identified the things in the video to which the facilitator directed attention

These elaborations of *what* ideas were being selected were coded into one of the following categories: teaching, teaching in interaction with students and the task, cognitive demand, students’ ideas, the factors, or other. A summary of these codes can be found in Additional file 2. This expanded coding of the moves which allowed us to identify the contributions made by the participants *and* the ideas emphasized by the facilitator. These second-layer codes were critical to our analysis because they provided a means by which to test whether the PD goals and subgoals were used to guide TSCD-PD discussions in productive directions.

None of the facilitator moves identified in the van Es framework was tightly related to the *connecting* practice, which is about connecting participant ideas to a larger idea related to teaching and learning. Thus, we coded facilitator moves related to the connecting practice separately. Specifically, we coded for the extent to which the facilitator drew *connections* between participants’ contributions and the overall goal: Instructional factors that can maintain or decline cognitive demand. Examining if and how facilitators made connections to this big idea was important because such connections play a critical role in elevating participants’ emerging understanding to a level of generalization that affords applicability beyond a particular lesson, task, or teacher. When a move was coded as “making a connection,” we further coded the connection by identifying (1) whether the facilitator and/or the participant(s) made the connection and (2) whether the participants’ connecting comments were best characterized as (a) *surfacing* more clear reasons/factors associated with maintenance or decline in cognitive demand by comparing and contrasting two video clips or (b) *labeling* by giving the factor a name that they generated and adding them to the list of factors associated with maintenance and decline, or trying to use the labels that they had added to the list of factors.

After mapping the correspondence between the van Es et al. framework and the *Five Practices* Framework (Table 2) and reading through several of the transcripts of the TSCD-PD sessions, the first author developed a codebook which both authors then used to code part of the PD-6 transcript. A discussion of their coding agreements and disagreements led to further refinement of the codebook. The first author coded the remainder of the transcripts in two rounds with further refinement of the description of the codes and the distinctions between them. Segments of the transcript that were challenging to code were discussed between the two authors.

The fact that the main PD facilitator was also the primary coder presents challenges to claims of objectivity. We worked to maintain objectivity in several ways (Bogdan & Biklen, 2003). The main avenue for data collection was videotaping, thus limiting the problem of working from field notes that potentially reflect observers’ biases (i.e., what is collected is influenced by what is noticed, what is focused on, and what is ignored). All of the video records—including the PD session videos that were analyzed for this paper and the teaching videos shown in the PD—were accompanied by transcripts, thereby making reference to specific details (that support or contradict) one’s claims possible. Finally, throughout the process, the authors worked as a team to check each other’s biases and to “acknowledge and take into account [our] own biases as a method of dealing with them” (Bogdan & Biklen, 2003, p.34). All in all, we recognized that our biases may have effected coding decisions but believed that the danger was offset by the depth of coding and analysis that was possible given the authors’ intimate knowledge of the program’s design and goals for teacher learning.

## Results

### Practice 3: *Monitoring* responses

During the lesson, teachers monitor students as they work in small groups exploring the task on their own before discussing it as a whole class. The teacher’s monitoring activity is viewed as helping them to get a handle on (a) how the class as a whole is doing and (b) which pieces of student work might be worth selecting for sharing with the whole class. We define *facilitator’s monitoring moves* as facilitator’s keeping an eye on participants’ thinking as they engage in the PD task. Depending on the size of the group, monitoring could happen either during small group work or during the whole group discussions. Since the TSCD-PD involved only five teachers, monitoring happened during the whole group discussions.^3^ Despite the fact that monitoring in the classroom is done during small group work, the TSCD-PD facilitator engaged in similar work by keeping track of how participants were thinking about the classroom interactions that they were observing in the video.

To examine the extent to which monitoring actually happened in the TSCD-PD, we analyzed each facilitator move in the transcripts of the TSCD-PD sessions. As shown in Table 2, we considered facilitators’ moves that were coded as “clarifying,” “countering,” “redirecting,” “distributing participation,” “linking participant ideas,” and “validating participant ideas” as evidence of facilitators’ paying close attention to what participants were (or were not) saying and, if needed, assuring that the discussion remains focused on the PD task at hand. Our analysis provided evidence that these monitoring moves were used in nearly all the TSCD-PD sessions (see Table 3).

**Table 3:** Frequency of monitoring moves across the TSCD-PD sessions

	PD2	PD3	PD4	PD5	PD6	PD7	Total
Total moves	*n* = 42	*n* = 137	*n* = 68	*n* = 100	*n* = 174	*n* = 101	
Clarifying	6	27	18	26	20	9	106
Distributing participation	3	11	4	9	21	5	53
Validating participant ideas	1	7	3	2	12	10	35
Countering	3	3	2	4	6	8	26
Redirecting	0	2	5	2	3	3	15
Linking participant ideas	1	0	2	3	7	2	15
Total monitoring moves	14	50	34	46	69	37	

The most frequently used monitoring move was *clarifying* which involved restating and revoicing to ensure common understanding of an idea. Also noticeable was the increase in *validating participants’ ideas* in the last two PD sessions. We suspect that this increase was spurred by that fact that participants were analyzing their own and each other’s video clips in these last two sessions and therefore might have needed more facilitator validation in order to feel comfortable sharing their ideas about their colleagues’ videos. van Es et al. (2014) stated that “when participants appeared to take risks and offer an interpretation of what they saw happening, statements such as these [validating comments] expressed support for their participation and encouraged further contributions” (p. 328).

### Practice 4: *Selecting* participant responses to share or highlight

Selecting pieces of student work to highlight is a critical practice for classroom teachers. If selected well, the pieces of student work will provide fodder for classroom discussion that moves the class as a whole toward the learning goal of the lesson. “Selecting,” done well, allows the teacher to both build on student thinking and lead learners to the canonical understanding that she wants them to leave the lesson with. Similarly, in PD, the *facilitator* can subtly steer the overall discussion toward the ideas she wants them to take away from the session (the goals or subgoals) by selecting certain participant contributions to highlight and particular pieces of video clips to draw participants’ attention to. Just as a teacher selects student work by its potential to bring out important ideas related (or “on the way”) to the learning goal, the PD facilitator can elevate certain things and not others as a way of scaffolding the learning of the group as a whole toward the identified goals for teachers’ learning.

To examine the extent to which this practice was actually adopted by the TSCD-PD facilitators, we examined the facilitator moves that were coded as highlighting, lifting up, or pressing under the van Es framework. These moves allow facilitators to choose certain ideas to discuss with the participants. Unlike classrooms in which actual (physical) pieces of student work are available for selection, TSCD-PD sessions did not produce physical pieces of participants’ work. Instead, we consider participants’ comments (as the tangible products of their thought processes) as the “items” that are available for “selection.” For example, the facilitator can choose to focus on particular comments that the participants make while gently disregarding others. Similarly, the facilitator can purposefully choose to bring participants’ attention to certain ideas illustrated in the video clip.

Like with the monitoring moves, our analysis provided evidence that selecting moves were used in nearly all the TSCD-PD sessions, but in comparion to monitoring moves, there were relatively lower frequency of selecting moves (see Table 4).

**Table 4:** Frequency of selecting moves across the TSCD-PD sessions

Total moves	PD2	PD3	PD4	PD5	PD6	PD7	Total
	*n* = 42	*n* = 137	*n* = 68	*n* = 100	*n* = 174	*n* = 101	
Pressing	6	21	6	12	18	7	69
Highlighting	1	3	0	3	19	7	36
Lifting up	3	14	1	3	4	2	27
Total selecting moves	10	38	7	18	41	11	

**Fig 2:**
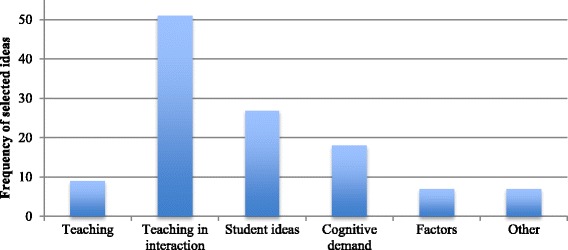
Substance of ideas that the facilitators selected

More importantly, the majority of the facilitators’ selecting moves were consistent with the goals of the TSCD-PD. Specifically, the facilitators mainly selected ideas related to teaching-in-interaction, cognitive demand, and student ideas. Figure 2 shows the overall distribution of ideas that the facilitators selected across all the PD sessions.

As shown in Fig. 2, majority of facilitators’ *selecting* moves focused on teaching-in-interaction and student ideas. This means that facilitators were frequently focusing participants’ attention on what and how students were thinking or the interaction of the teacher and students around the ideas embedded in the task. For example, in session 6, the facilitator invited participants to discuss “teaching-in-interaction” by pointing to a particular line in the transcript of the video to *highlight* a debate between the students and the teacher as the students were trying to make sense of the data in the task:

Facilitator: So I want to bring attention to this heated debate, to what the students were saying, and then specifically about Ms. [Nancy] Smith’s (the teacher in the video) questioning; I just want to go back to this heated debate part. So I think it starts—let’s start from line 70…

As illustrated in this excerpt, the facilitator invited participants to focus not only on what students were saying but also on what the teacher did in relation to what students were saying (i.e., the questions that the teacher asked in relation to students’ ideas).

As expected, facilitators also elevated participants’ ideas about cognitive demand. For example, in session 3, participants were discussing whether the cognitive demand of the task was maintained during its implementation, which was shown in the video:

Linda: Oh. She probably increased the cognitive demand.

Susan: There’s no guidance whatsoever.

John: Right.

Barbara: I said, the teacher did not give much direction, was my first comment.

*PD participants were asked to first write their reactions during private think time before the discussion began.*

Carol: Well, she guided them. She just didn’t guide them through the worksheet.

Facilitator: So first, Ms. [Linda] Williams, say more about what you mean, that she increased the cognitive demand.

In the above example, the facilitator took up Ms. William’s comment about cognitive demand and made it the object of the discussion by asking Ms. William to expand on what she said. Facilitators also *pressed* participants to say more about their ideas related to cognitive demand throughout the PD sessions. Pressing is used to “prompt participants to explain their reasoning and/or elaborate on their ideas” (van Es et al., p. 347). The following excerpt illustrates how the facilitator prompted participants to say more about their categorization of the level and kind of student thinking in the video based on the categories of the TAGS:

Carol: I said low/high. I gave the first one a 2 [cognitive demand of tasks involving scripts]

Facilitator: Which 2?

Carol said she categorized the level and kind of student thinking in the video into level 2 based on the TAGS. However, level 2 of the TAGS consists of three categories depending on whether or not students are positioned to engage in scientific practices. Thus, to invite participants to think about the cognitive demand level in more detail, the facilitator asked Carol which level 2 category of the TAGS she was referring to.

As shown in Fig. 2, facilitators also selected participants’ ideas related to the instructional factors associated with maintenance and decline. For example, one of the instructional factors associated with maintenance that was labeled in session 6 was teacher questioning. Then, in session 7, as participants were analyzing a co-participant’s classroom video, Nancy commented on her questioning:

Nancy: I thought Ms. [Linda] Williams asked them nice questions.

Facilitator: Yeah, yeah. This is why I want to look at her questions, because –

Nancy: Well, I wrote them all down.

Facilitator: —if you can—I mean, in this classroom, I think we’re all in agreement that it was all about like “Why?” “What does it mean?” “Tell me what two bands mean.”

Carol: Literally, she just kept saying, “What does it mean?”

Facilitator: But something was not working. I just want to talk about it. So she was like pushing them and—

Nancy: Well, I wrote she pushed them in a few different spots with their own line of thinking.

Facilitator: So let’s look at some of the questions that she was asking ____ –

In this classroom video excerpt, Ms. William was repeatedly asking the same questions instead of tailoring her questions to how individual students were thinking. After hearing Nancy’s comment on Ms. William’s questioning, the facilitator invited participants to think harder about her questions, specifically in relation to what students were saying and how they were thinking about the task. In short, the facilitator lifted up Nancy’s comments on Ms. William’s questions and invited participants to analyze Ms. William’s questions in more detail because there was something to learn there about the factor related to questioning.

All in all, our detailed coding of the facilitators’ highlighting, lifting up, and pressing moves indicates that facilitators were selecting ideas for further discussion to advance participants’ understanding toward the goals of the TSCD-PD. Specifically, facilitators tried to focus attention on participant comments about teaching-in-interaction with the students and the task, making sense of the cognitive demand in the classroom videos and identifying factors associated with maintenance and decline. Pressing for student thinking was also prevalent across the PD sessions, which is not surprising given that participants made decisions about the level and kind of student thinking by closely making sense of students’ ideas. Therefore, in addition to focusing on teaching-in-interaction, participants,not surprisingly, needed to discuss students’ sensemaking. This finding is consistent with the recent studies, which emphasize supporting teachers’ learning to make sense of students’ thinking (e.g., Sherin & Han, 2004; Levin & Richards, 2011; Levin et al. 2009; van Es & Sherin, 2007).

### Practice 5: *Connecting* participants’ responses to the big idea

Ultimately, teachers need to recognize classroom interactions as instances of larger patterns that represent generalizations that can be useful for guiding interpretations and actions instead of treating each interaction separately, as if it was being encountered for the first time. Connecting to “big ideas” can be useful in that regard because they provide an overarching framework within which to view one’s own teaching behavior. A teacher may, for example, start to notice that she is turning an open-ended problem into a step-by-step exercise and that students are happily following directions without understanding.

Similarly, an overarching role of the facilitator, we argue, is to draw connections to these big ideas so that teachers take away not only isolated instances of teaching and learning but also a larger framework of big ideas with several specific instantiations of what each component looks like in practice. To understand the extent to which this actually happened in the TSCD-PD, we examined the facilitator’s moves and participants’ contributions with regards to the kind of connections that were made. Surfacing how they made connections to a big idea allowed us to elevate participants’ emerging understanding to a level of generalization that affords applicability beyond a particular lesson, task, or teacher.

**Fig. 3:**
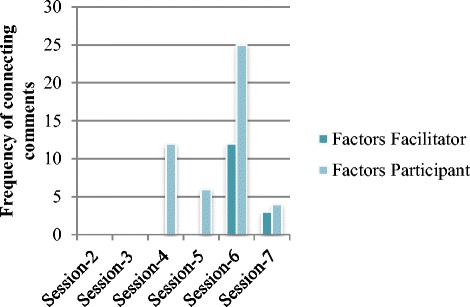
Change in the frequency of connecting to factors comment across the PD sessions

Figure 3 provides the frequencies of facilitators’ and participants’ comments in which they made connections to the factors. As shown in the figure, facilitators and participants made more connections to factors in the later PD sessions. The following excerpt illustrates an instance of the facilitator co-constructing^4^ a factor (asking students to support their explanations with evidence) with the participants as they were analyzing a video from a co-participant’s classroom.

Susan: I just wondered like if you (speaking directly to the co-participant featured in the video) had just took the observation. That’s how I handled it (referring to when she taught the same lesson), so (tell the students) “just look and *observe* right now. And then...”

Carol: Oh, no, I had them do both [observations and interpretations of the data].

Susan: Both at the same time?

Carol: Yeah.

Susan: Okay.

Researcher: Which I think worked better. At least at that level.

Linda: Well, they all seemed to want to start with a rule, and then explain it.

Carol: I pretty much just told them, whatever you tell me, you have to be able to prove it.

Linda: Right.

Carol: So with that, they had to do both (they had to make observations of the data and interpret those observations).

Facilitator: So can we add this to our list of maintenance factors? Asking for evidence for students’ claims or arguments or explanations? Do you think that it helped to maintain cognitive demand?

Linda: I—well, I said—I said always keep them engaged in the scientific practice. Is that the same thought (the same factor)? Like to maintain a lesson, you have to keep them focused on the practices as well?

As illustrated in this excerpt, participants were discussing an instructional practice that Carol had adopted—requesting that students provide evidence for their claims—which they thought was influential in students’ making sense of the data. The facilitator suggested adding this instructional practice to the list of factors that they were generating on chart paper. Then, the discussion continued with Linda’s comment about whether one of the existing factors in the list (i.e., engaging students in scientific practices) already included this idea of asking students for evidence for their claims. So, they were negotiating what factor to add to the list and how to best represent it.

Our analysis also revealed a change over time in participants’ comments related to the factors of maintenance and decline across the TSCD-PD sessions. The nature of their comments differed qualitatively from session to session, following the trajectory of *sensing* teaching factors that might be playing an important role in what and how students were thinking, to *surfacing* specific instances of teaching practices that impacted student thinking, and to *labeling* them and trying to use the labeled factors in their analysis of their own videos.

In sessions 2 and 3, participants began to “sense” that teachers could shape the level of student thinking. Their comments were unsophisticated involving general—even vague—terms. Remember, the participants have not yet been introduced to the idea that the cognitive demand of high-level tasks could be maintained or declined or to the instructional factors. That is why these comments were not coded as “connection to factors.” Nevertheless, we were able to identify comments which reflected the participants’ beginning to *sense* instructional factors associated with maintenance and decline. For example, in session 2, one of the PD participants said:

Participant: Yeah, that’s what I wrote. Her (the teacher in the video) questions are all statements. She did everything so they technically could just sit back and chill.

Facilitator: Ah, okay, so you are saying that students can just sit back and then not do anything—ah, she could—.

Participant: Because of how she presented it, she didn’t put anything—she did all the work. She didn’t put anything on them.

As this excerpt illustrates, the PD participant recognized how the questions/statements that the teacher in the video asked influenced the students’ thought processes. She described that the teacher did all the thinking work for the students. Similarly, in session 3, Carol said, “So like if you ask them the question and almost give them the answer, you can take the thinking away from the kids and think for them. That’s sort of what she (the teacher in the video) was doing.” Again, Carol explained, using her own words, how the teacher in the video lowered the demand on students’ thinking and reasoning.

The nature of participants’ comments related to factors began to change in session 4. This was not surprising because, as we discussed earlier, in the beginning of session 4, participants were introduced to the idea that cognitive demand can decline during the implementation of a high-level task. After this, participants began viewing the videos through this new lens.

In particular, while analyzing the contrasting video cases in session 4, their “connecting comments” began to *surface* specific instructional factors with an explicit reference to the idea of why cognitive demand might have been maintained or declined. In this session, one video illustrated a high-level task that was proceduralized by the teacher; in the second video (which featured the implementation of the same high-level task), the teacher focused on helping students understand the underlying biological idea behind the modeling task about genetics. Recognizing the differences, Linda said:

…I have two thoughts right now, that the first teacher (in the video) kind of turned this totally into a Punnett square because there were a couple examples of this. The very first group did fertilization and didn’t have alleles in their egg and sperm. She (the teacher) didn’t make them pull them apart. She made them add the alleles.… it went against that biological concept of the alleles don’t come after or don’t combine. So she didn’t do the meiosis part. Second piece of evidence was once she started doing “this” and “these” with all those groups, she just kept saying, “How many combinations did you get? How many combinations did you get? How many combinations did you get?” Or, “How many combinations can you get?” And they were like, “Oh, let’s do it. Okay, hurry up.” And I even saw that one kid go, “Come on, let’s roll with it,” and he just started sticking stuff and they started combining. It was all about the combinations for her, how many combinations—

As illustrated in this excerpt, Linda surfaced how proceduralizing the task and focusing on the end result could influence the level and kind of student thinking. Similarly, in session 5, participants continued to surface instructional factors associated with maintaining or declining cognitive demand without naming them factors. For example, when contrasting the two videos, Barbara said:

Only because I felt like the teacher was doing all of the cognitive work, and just kind of pulling like words, instead of really saying, well, “What are you trying to tell me? What do you mean by that?” They were asking questions, she was answering it. It was that back and forth. And then in the second video I said it was a high level, and level 4, only because I think the small group work really helped. I think if it was just the classwork, maybe she wouldn’t have been able to do that. I think adding that small group work really brought it up, because then students were actually looking at data, analyzing data, making observations, comparing data between different results from different crosses. They were struggling with the information, so I thought there was a high cognitive demand, because, you know, they were really trying—all of them really started to notice, well, all of these PCRs and all of these western blots are the same. Why don’t they all look the same? So they’re really struggling, trying to figure that out. So I thought that that made it high level. And the teacher wasn’t just answering the questions.

During her discussion of the contrast between the videos, Barbara was surfacing factors that could play a role in determining the level and kind of student thinking that occurs in response to a task. For example, she uncovered that in one video, the teacher was directly answering the questions that students asked, instead of asking follow-up questions to elicit students’ thinking. The nature of the intellectual work that the students engaged in was, then, different in these two classrooms.

Finally, in the last two sessions, participants analyzed videos from their *own* classrooms. In the beginning of session 6, the facilitator engaged participants in a “summary activity” during which she introduced the idea of factors to the participants as they were summarizing what they had done in the prior sessions with the contrasting video cases. Then, the facilitator said:

So when we talked about the differences, we actually talked about the factors that cause maintenance or decline of the task, actually. But we did not call them factors, but you started to call them factors about what causes maintenance or decline. So, let’s call them factors associated with maintenance or decline…

During this summary activity, participants began to list factors associated with maintenance and decline, factors that were brought up (mostly) during the discussion of the contrasting video cases. Initially, the participants shared things like classroom management, student absenteeism, and class and/or group size as factors associated with the decline in cognitive demand. The facilitator added these factors to the list but then gently invited them to think about factors that are more under their control. The discussion then started to move toward developing a list of instructional factors, such as asking open-ended questions and eliciting student thinking. For example, Susan suggested that teacher-questioning was a factor associated with maintenance and decline. She said:

Susan: I think the method of questioning, if that makes sense.

Facilitator: So say more

Susan: If I were going to do that task where you give them the flowers, which is what that video was, right? So one way to get them to realize the different offspring would be like, okay, so let’s look at the first one. It’s red and it’s white. What do you notice about the offspring? They’d be like, oh, it’s red. Okay. How is that different than all the rest? That would be a decline, because you’re really direct—like this is point blank the easy answer.

After generating this list of factors, the participants started to discuss the video clips from their own classrooms. During their discussions, they often referred to the instructional factors listed on the chart board and they sometimes added new ones to the list. For example, the following excerpt illustrates Susan’s attempt to use the factors of maintenance as they were analyzing Nancy’s video in session 6:

I think that you were asking questions for them to clarify, and allowing them to work through the problem. So that one heated-debate group, you allowed them to work through it. And then also that the students were developing the rules on their own. Like even you say, so you’re saying, and then you go into a repeat back of what they’re saying to clarify. What does that mean on here? So you’re kind of like pushing them to explain it. And then even like (line) 137, the student says, now you just match them up, and you say, what? What are you matching? And you’re pushing them to like clarify it that way.

As illustrated in this excerpt, Susan was sharing her observation of the video by connecting teacher’s action’s to a broader instructional factor associated with the maintenance of cognitive demand. One of the maintenance factors listed on the chart paper was “Questions: Clarification questions, open-ended, making thinking visible for students.” Susan was clearly trying to use this language to explain how the teacher was making students “work through” the task by asking clarification questions.

All in all, our analysis of facilitators’ and participants’ connecting comments suggested that TSCD video-based PD fostered participants’ capacity to make connections to the bigger ideas of teaching science by maintaining the cognitive demand on students’ thinking.

## Discussion and Conclusions

The new vision for the next generation of science teaching and learning will require increased capacity on the part of teachers. One of their biggest challenges will be to learn to enact new, high-demand tasks in ways that engage students with the scientific concepts while, at the same time, engaging them in the science practices. As with other transformative reforms, science teachers will need to participate in carefully designed professional development to help them learn how to successfully support their students’ learning using complex tasks.

Professional development that is centered around viewing and discussing classroom videos is an increasingly recommended approach for supporting teachers’ capacity to enact the vision of NGSS. Like any professional development, however, video-based PD is not self-enacting. It will succeed or fail based on how PD providers plan for and facilitate experiences that are built around videos.

Despite being called “a new cadre of prominent players on the educational scene” (Borko et al., 2014b, p. 149), we know little about how PD providers go about their work. The need to understand what they do behind the scenes as they design learning experiences for teachers and how they facilitate discussions around video is becoming more urgent as the numbers of video-based PDs increase. However, there is an admittedly thin research base focusing on the facilitators of PD programs and the knowledge and skills that they need (e.g., Borko et al. 2014a; Elliott et al. 2009; Jackson et al. 2015; Koellner, Jacobs, & Borko, 2011; Lesseig et al. 2016).

Our use of the Five Practices Framework as a tool for examining the work of PD facilitators is a step in the direction of addressing this gap in the literature. Although developed for a different purpose (math and science classrooms), the broad-based alignment between preparing for and enacting a successful lesson and designing and facilitating a PD session (both are teaching and learning situations around complex tasks) allowed us to make necessary adaptations fairly easily. In short, we found that using the framework to guide our inquiry was possible. But what has it enabled us to see that otherwise we may not have and what, if anything, has it obscured?

The practice of setting goals turned out to be a surprisingly powerful lens through which to view the design and enactment of video-based PD. The initial detailed specification of goals for the TSCD-PD provided a host of benefits downstream. For the facilitators, the goals and subgoals provided guidance for the design of each session. For the researchers, the goals and subgoals became a way to monitor if discussions were moving in a productive direction or if they had become a “free for all.”

Most powerful, however, was the examination of the facilitators’ *combined* use of goals and the “selecting practice” during the actual discussions. Using both to interpret facilitator moves revealed facilitator choices—made mid-stream during discussions—that otherwise would have remained invisible. By illuminating not only the fact that the facilitator “pressed” or “lifted up” certain participant comments but also coded *what* the essence of those comments were, we witnessed deliberate choices on the part of the facilitator to pay attention to teachers’ thinking *and* to move their thinking toward the goals/subgoals of TSCD-PD (and not to pursue other topics). Attending to teachers’ thinking is consistent with other researchers’ recognition of the need for facilitators to learn how to notice teacher thinking (Borko et al., 2014a; van Es, 2010). With the Five Practices Framework as our guide, however, we were able to also uncover the need for facilitators to know what to do with the teacher ideas that they noticed. Specifically, selecting and connecting practices can help facilitators to support teachers’ learning toward carefully defined big ideas of teaching without undermining their sense of agency.

Equally compelling was the trajectory of participants’ learning along a pathway that was also “designed into” the plan of the seven-session experience. The trajectory from sensing, to surfacing, and to labeling essentially was not fully formed from the beginning. What was fully formed was a commitment to an approach to learning that first set up learner-led exploratory experiences, followed by experiences that channeled that exploration a bit, to a culminating experience in which the learning is directly addressed by the teacher/facilitator and connected to learners’ initial less-structured experiences. Consistent with that theory, we (the authors of this paper) did not (indeed we could not) “label” the three phases of sensing, surfacing, and labeling until we began to reflect on the experiences and the data. It was only then that we realized how the sessions combined elements of participants’ grappling to make sense of videos with solidifying their understandings. Treating teacher learning as a progression has recently been emphasized by others as well. Jackson et al. (2015) examined mathematics leaders’ capacity to support teachers’ learning across a large US school district. One of their goals for mathematics leaders’ learning was treating teacher learning as a progression, and their mathematics leaders began to achieve this over time. They began to conceptualize the PD sessions as a sequence of linked activities instead of disjointed actvities.

We think that the sensing, surfacing, and labeling pathway can be used by others to both design PD and to evaluate teachers’ learning progressions in PD experiences. It would be especially useful to apply to the study of how participants might grow and develop over the course of an extended, multi-session professional development experience. The design allowed for large stretches of participant-generated understandings gained through grappling with complex features in the video clips. However, there were also times for “telling,” as when the facilitator expounded on the “journey of a task” or factors associated with maintenance and decline. Both forms of learning can be valuable, but especially when they occur in the order in which they were *sequenced*: Grappling with challenging ideas, followed by a consolidation that builds on the emerging understandings rather than being espoused or proclaimed from the start. The way the sequencing practices were adopted by the TSCD-PD designers/facilitators provides a concrete illustration of how teachers’ learning experiences can be sequenced within the PD sessions and more importantly across the PD sessions by approaching teacher learning as a progression.

We see our findings as demonstrating that it is feasible to carefully design *and execute* a sequence of PD sessions around the medium of video. Considered in tandem with previous studies on TSCD-PD, we also see our findings as endorsing the promise of goal-driven, theory-informed design that forefronts careful attention to teachers’ thinking to support their understanding of complex classroom interactions and ambitious instructional practices. Future work will involve implementing TSCD-PD in additional sites with different facilitators (moving on to what Borko (2004) has referred to as phase 2 of research on professional development).

## Additional files

Additional file 1: An example instructional task from the Design Unit. (PDF 161 kb)

Additional file 2: Categories for coding the substance of participant ideas selected by the TSCD-PD facilitators. (DOCX 111 kb)

## Footnotes

1 We use the term “participants” to refer to the teachers who were in the TSCD video-based PD. We reserve the term “teachers” for the teachers in the video clips.

2 In addition to the TSCD-PD, these same teachers had spent approximately 6 h with the designers of the Design Unit, the vast majority of which focused on uncovering the genetic content in the Design Unit tasks. TSCD facilitators were participant observers in those larger project PD sessions without any facilitation role.

3 We did not have a designated time period during which participants worked in small groups on a particular PD task. However, this could be the case in some PD, which involves larger number of teachers. In such cases, it may be better to do monitoring before whole group discussions with the teachers.

4 It is important to note that some of the facilitator’s comments that connected to the factors in sessions 6 and 7 were in fact co-constructed by participants and the facilitator together. In these comments, participants made contributions to several of the “connecting comments” by the facilitator or facilitator’s “connecting comments” originated from what the participants said.
