# Supplementary material for: A framework for planning and facilitating video-based professional development
Source: Int J STEM Educ. 2017 Nov 21;4(1):28. doi: 10.1186/s40594-017-0086-z (PMC6310388; doi:10.1186/s40594-017-0086-z)
Supplement: Supplementary file 1 — An example instructional task from the Design Unit. (PDF 161 kb) [file 40594_2017_86_MOESM1_ESM.pdf]

Name________________________________**Teacher**_______________________**Date**____________

Understanding Inheritance Worksheet 3A: Exploration of Mating Results

Using a technique called polymerase chain reaction, scientists can see what is happening in organisms at the DNA level. The PCR results depicted below show variation in DNA for the same gene. On the picture below, circle the level which is being addressed in this worksheet.

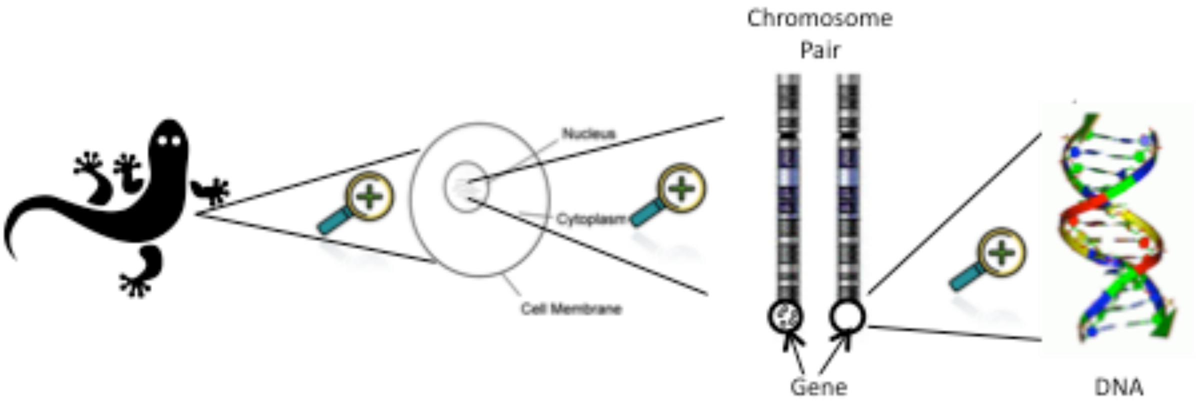

Study the results of the two crosses which are shown below. What observations can you make?

Cross 1

The results of Cross 1 for Male 1 and Female 1 as shown on a computer printout.

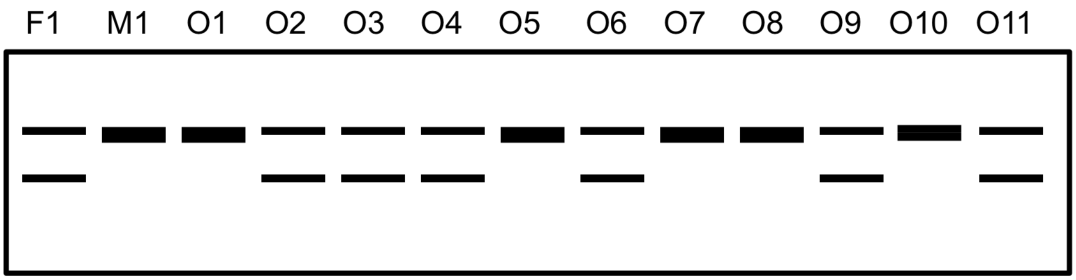

Key: F1=Female 1
M1 = Male 1
O1-11=Offspring

Cross 2

The results of Cross2 for Male 1 (same Male as in Cross 1) and Female 2 as shown on a computer printout.

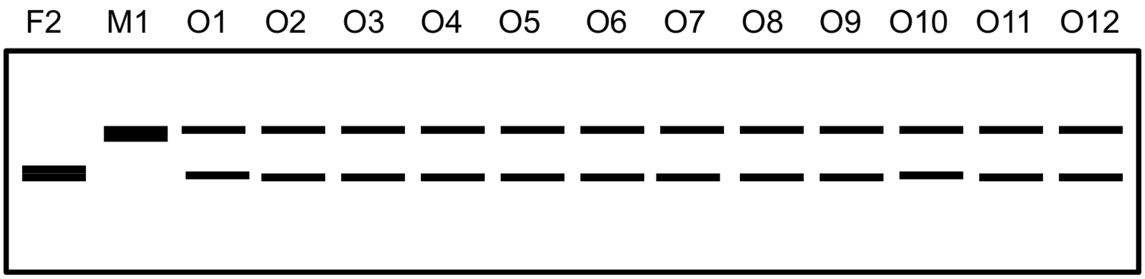

Key: F2 = Female 2
M1= Male 1
O1-O12 = Offspring

Please write your observations in the space below. You can use the back of the sheet if needed

Name______________________________Teacher______________________Date____________

Understanding Inheritance Worksheet 3A: Exploration of Mating Results

Based on your observations, what are some general rules that explain the types of offspring seen in cross 1 and cross 2 and why they differ from one another? Please support your rules with evidence (observations from the cases). Remember the rules should apply to both cases.

**Rule**	**Evidence/Observations**
