# Supplementary material for: A framework for planning and facilitating video-based professional development
Source: Int J STEM Educ. 2017 Nov 21;4(1):28. doi: 10.1186/s40594-017-0086-z (PMC6310388; doi:10.1186/s40594-017-0086-z)
Supplement: Supplementary file 2 — Categories for coding the substance of participant ideas selected by the TSCD-PD facilitators. (DOCX 111 kb) [file 40594_2017_86_MOESM2_ESM.docx]

The Substance of participant ideas selected by the TCD-PD facilitators

| Code | Definition | Example |
| --- | --- | --- |
| Teaching | Ideas that focused on the teacher’s instructional practices, which were not grounded in students’ thinking | Nancy: The similarity, I had that the teacher had gone to all the groups, but also I kind of generalized that too as leading hints. Both teachers gave –  Rema: I think I wrote that too.  Nancy: I don’t know the correct word to use like  Linda: Guided questions.  Nancy: Guided questions.  Rema: This says they pushed the kids towards the correct answer.  Nancy: Yeah.  Facilitator: Both of the teachers push students? *(Facilitator lifted up an idea that focuses on what the teachers did and is independent of students’ ideas)*  Rema: Towards the correct answer. Some of them literally carry the kids here, like common kids. |
| Teaching in interaction | Interactions of the teacher and students around an idea embedded in the task | Facilitator: … So she [the student] kind of uses the idea of “offspring get DNA from – ” and then the teacher says, “offspring gets what?”, and then the student says, “DNA from their parents”. And then “what are we talking about ____ specific”, the teacher says, and then the student says “traits”, and then teacher says, “sort of”. And then student says, “DNA”. Then, teacher says, “You are technically right. They get their DNA from – ”and then student this time says “characteristics”. So she keeps saying – first she started with DNA, continued with trait, now she's saying characteristics. And then how did Carol [the teacher] respond? *(Facilitator highlighted the interaction between the teacher and students about the ideas embedded in the task and then she asked participants to think about how the teacher responded to students’ ideas)* |
| Cognitive demand | Comments about the level and kind of student thinking in the videos and whether the cognitive demand was maintained or declined during the set-up or implementation of the task | Facilitator: Okay. So what were the other features of level 4 that you see in these videos? *(The facilitator pressed participants to provide evidence from the video for their categorization of level of student thinking into level-4 based on the TAGS)* |
| Student ideas | What students appeared to be thinking about the science ideas embedded in the task | Barbara: Or she says a dominant trait on 135.  Linda: What do you mean by straight? Line 74.  Facilitator: What does he mean with “straight” really? *(The facilitator lifted up what Linda said about the student’s comment to have them elaborate on student thinking)* |
| Factor | Instructional practices/moves associated with maintenance and decline of cognitive demand | Facilitator: What were some other factors, we discussed in the last two PDs when we compared the two videos that caused either maintenance or decline in the classrooms that we observed – I mean, that we viewed?  Susan: I think the like method of questioning, if that makes sense.  Facilitator: So say more. *(The facilitator pressed the participant to elaborate on one of the instructional factors that she identified as important for maintenance and decline of demand on student thinking)* |
